# Supplementary material for: A stochastic vs deterministic perspective on the timing of cellular events
Source: Nat Commun. 2024 Jun 20;15:5286. doi: 10.1038/s41467-024-49624-z (PMC11190182; doi:10.1038/s41467-024-49624-z)
Supplement: Supplementary file 1 — Supplementary Information [file 41467_2024_49624_MOESM1_ESM.pdf]

Supplementary material for
A stochastic vs deterministic perspective on the timing of cellular events

Lucy Ham[1,2,†], Megan A. Coomer[1,2,†], Kaan Öcal[3], Ramon Grima[4], Michael P.H. Stumpf[1,2,*]

[1]*School of BioSciences, University of Melbourne, Parkville VIC 3010, Australia*
[2]*School of Mathematics and Statistics, University of Melbourne, Parkville VIC 3010, Australia*
[3]*School of Informatics, University of Edinburgh, Edinburgh, EH8 9AB, United Kingdom*
[4]*School of Biological Sciences, University of Edinburgh, Edinburgh, EH9 3JH, United Kingdom*

Abstract

Here we provide details and proofs of the results given in *The timing of cellular events: a stochastic vs deterministic perspective.*

Supplementary Note 1 - Computing moments of the FPT distribution

In the main text, we derive moments of the FPT distribution for any continuous-time Markov process $\mathbf{x}(t)$. We here illustrate our approach by way of a simple example. Consider a simple birth-death process with transcription rate K and degradation rate δ,

$$\varnothing \underset{\delta}{\overset{K}{\rightleftharpoons}} P. \tag{1}$$

Here P represents protein. An illustration of the simple birth-death process is given in Figure 1(A). The state of the system, S, is the set of non-negative integers, $S = \{0, 1, 2, \dots\}$. Fix some target protein number $N \in S$, and suppose we are interested in the average time that it takes for the birth-death process to reach N given that the system is started in state $n \leq N$. In other words, we are interested in finding,

$$\mathbb{E}[\tau_n] = \mathbb{E}[\inf\{t \geq 0 \text{ such that } X(t) = N\} |\ X(0) = n].$$

To find $\mathbb{E}[\tau_n]$, we begin by finding an expression for the average time that it takes to reach N from n, conditional on the next state, which we will denote by n'. Letting $D_n := n\delta$ and $R_n := n\delta + K$, we may observe some simple linear relationships between neighbouring values of $\mathbb{E}[\tau_n]$. From a value of n, there are just two neighbouring states $n' = n+1$ (birth) or $n' = n-1$ (death). And these occur with birth and death probabilities K/R_n and D_n/R_n, respectively. The time that it takes to get from n to N is the time that it takes to get from n to n' plus the time that it takes to get from n' to N. Hence the expectation of the time taken to get to N from state n, given the system transitions to n', is $R_n^{-1} + \mathbb{E}[\tau_{n'}]$. It then follows from the Law of Total Expectation that,

$$\mathbb{E}[\tau_n] = R_n^{-1}\left[D_n(R_n^{-1} + \mathbb{E}[\tau_{n-1}]) + K(R_n^{-1} + \mathbb{E}[\tau_{n+1}])\right].$$

Rearranging, we obtain the tridiagonal recurrence relation,

$$R_n\mathbb{E}[\tau_n] - D_n\mathbb{E}[\tau_{n-1}] - K\mathbb{E}[\tau_{n+1}] = 1, \tag{2}$$

*Correspondence: mstumpf@unimelb.edu.au
†These authors contributed equally to this work

with $\mathbb{E}[\tau_N] = 0$. We impose $\tau_n = 0$ for $n < 0$ so that the recurrence relation is valid for $n \in \{0, 1, \ldots, N-1\}$. Taking this set as our state space S_N can write Equation (2) in matrix form as,

$$\mathbf{A}\mathbb{E}(\boldsymbol{\tau}) = \mathbf{1}, \tag{3}$$

where $\mathbf{A}$ is an $N \times N$ tridiagonal matrix defined by,

$$\mathbf{A}_{ij} := \begin{cases} R_i, & \text{for } i = j \\ -D_i, & \text{for } j = i+1 \\ -K, & \text{for } j = i-1 \\ 0, & \text{Otherwise.} \end{cases} \tag{4}$$

Here $\mathbf{1} = [1, 1, \ldots, 1]^T$ is a vector of N 1's, and $\mathbb{E}(\boldsymbol{\tau}) = [\mathbb{E}[\tau_0], \ldots, \mathbb{E}[\tau_{N-1}]]^T$ is the unknown vector of mean first passage times. Equation (3) constitutes a finite system of linear equations which can solved numerically, which is the basis of our modified FSP approach (see subsection **??** above), or analytically in some cases (see Section 4 below).

Relationship to the Backward Master Equation

In the main text, we derive moments of the FPT distribution of arbitrary order (Equation (7) of the main text). We here demonstrate how our result agrees with that obtained from the Backwards Chemical Master Equation (BCME). We begin by observing the standard relationships between the FPT distribution, $f_{\boldsymbol{n}}(t) = P(\tau_{\boldsymbol{n}} = t)$, the cumulative distribution, $F_{\boldsymbol{n}}(t) = P(\tau_{\boldsymbol{n}} \leq t)$, and the survival function, $S_{\boldsymbol{n}}(t) = P(\tau_{\boldsymbol{n}} > t)$,

$$F_{\mathbf{n}}(t) = \int_0^t f_{\mathbf{n}}(s)\ ds \quad \text{and} \quad S_{\boldsymbol{n}}(t) = 1 - F_{\mathbf{n}}(t). \tag{5}$$

We thus have that,

$$f_{\mathbf{n}}(t) = -d_t S_{\mathbf{n}}(t). \tag{6}$$

It can be shown that the survival functions, $S_{\mathbf{n}}$, for all initial states $\mathbf{n} \notin \mathbf{Y}$, evolve jointly according to the backward CME on a truncated state space,

$$d_t \mathbf{S}(t) = \mathbf{A}_{\mathbf{Y}}^T \mathbf{S}(t), \tag{7}$$

where $\mathbf{S}(t)$ is the vector whose i^{th} component is $S_{\mathbf{i}}(t)$, and $\mathbf{A}_{\mathbf{Y}}$ is the state transition matrix of the CME for the modified system where every state in $\mathbf{Y}$ is absorbing. Taking another time derivative, and using Equation (6), the FPT distributions $\mathbf{f}_{\mathbf{n}}$, for all $\mathbf{n} \notin \mathbf{Y}$, can be seen to also satisfy the backward CME,

$$d_t \mathbf{f}(t) = \mathbf{A}_{\mathbf{Y}}^T \mathbf{f}(t), \tag{8}$$

where $\mathbf{f}(t)$ is the vector whose i^{th} component is $f_{\mathbf{i}}(t)$. The raw moments of the first passage time distribution,

$$\mathbb{E}(\tau_{\mathbf{n}}^k) := \int_0^\infty t^k \mathbf{f}_{\mathbf{n}}(t)\ dt, \tag{9}$$

can be computed by applying the backward master operator $\mathbf{A}_{\mathbf{Y}}^T$ to both sides of Equation (9),

$$\begin{aligned}[(\mathbf{A}_{\mathbf{Y}}^T)^k \mathbb{E}(\boldsymbol{\tau}^k)]_{\mathbf{n}} &= \int_0^\infty t^k [(\mathbf{A}_{\mathbf{Y}}^T)^k \mathbf{f}(t)]_{\mathbf{n}}\ dt \\ &= \int_0^\infty t^k d_t^k f_{\mathbf{n}}(t)\ dt \\ &= k!(-1)^k.\end{aligned} \tag{10}$$

Here we integrated by parts k times in the last step and used the fact that $\int_0^\infty d_t S_{\mathbf{n}}(t)\ dt = -1$. We can write this concisely as,

$$(\mathbf{A}_{\mathbf{Y}}^T)^k \mathbb{E}[\boldsymbol{\tau}^k] = k!(-1)^k \mathbf{1}. \tag{11}$$

We can solve for $\mathbb{E}[\boldsymbol{\tau}^k]$ provided that the matrix $\boldsymbol{A}_{\boldsymbol{Y}}^T$ is invertible. This occurs precisely when all initial states have a nonzero probability of reaching $\boldsymbol{Y}$; in this case all states will eventually reach $\boldsymbol{Y}$ and therefore $\tau_{\boldsymbol{n}} < \infty$ with probability 1.

The time-dependent FPT distribution

Using Equations (6) and (7) above, the FPT distribution $f_{\boldsymbol{n}}(t)$ can be recovered as

$$f_{\boldsymbol{n}}(t) = -\frac{\mathrm{d}}{\mathrm{d}t}\boldsymbol{S}_{\boldsymbol{n}}(t) = -(\boldsymbol{A}_{\boldsymbol{Y}}^T \boldsymbol{S}(t))_{\boldsymbol{n}}, \tag{12}$$

or, in more compact vector notation,

$$f_{\boldsymbol{n}}(t) = -\boldsymbol{A}_{\boldsymbol{Y}}^T \exp(t\boldsymbol{A}_{\boldsymbol{Y}}^T)\mathbf{1}, \tag{13}$$

where the entry $f_{\boldsymbol{n}}(t)$ is the probability density function of $\tau_{\boldsymbol{n}}$. The probability density functions, for all initial states $\mathbf{n} \notin \mathbf{Y}$, can be computed numerically by solving Equation (7) and storing the computed time derivatives at each numerical integration step. Julia's `DifferentialEquations.jl` provides functionality to access time derivatives of the solution, with interpolation. Importantly, we can compute the first passage time distributions for all initial states in one go by using the backward form of the CME.

Supplementary Note 2 - Analytical solutions of mean first-passage times for general birth-death process

In this section, we derive analytical solutions of mean first-passage times (MFPTs) for general birth-death processes, where the rates of births and deaths at any given time depends on how many extant molecules there are. This approach uses standard transformation techniques for reducing the order of recurrence relations, and can be found in [1]. We apply this general procedure to derive explicit solutions to three examples of biological relevance: (1) a simple birth-death process, (2) a reduced model of a genetic feedback loop, and (3) a birth-death process with Michaelis-Menten (MM) degradation. Note that examples (1) and (2) appear in the main text, while (3) is used below in Section 4.

In the following subsections, we will use the simplified notation τ_n^N to denote the expected waiting time to reach copy number N starting from $n \leq N$, and we will use τ_N^n to denote the expected waiting time to reach copy number N starting from $n \geq N$. This notation should not be confused with the Nth moment of the waiting time distribution.

The general procedure

Consider a general birth-death process with reaction propensities for birth and death given by $a^+(n)$ and $a^-(n)$, respectively. Here n represents the number of molecules in the system. It follows from Equation (11) (or Equation (7) of the main text) that the expected waiting time to reach N starting from an initial number of molecules $n \leq N$, satisfies the following recurrence relation,

$$(a^+(n) + a^-(n))\tau_n^N - a^-(n)\tau_{n-1}^N - a^+(n)\tau_{n+1}^N = 1, \tag{14}$$

with $\tau_N^N = 0$. There is no need to write special boundary conditions for these equations provided we impose $\tau_n = 0$ for $n < 0$. We now observe that the recurrence equation (14) can be re-expressed in terms of the forward discrete derivative of the sequence $(\tau_n^N)_n$ as follows:

$$a^-(n)(\Delta\tau_{n-1}^N) - a^+(n)(\Delta\tau_n^N) = 1, \tag{15}$$

where $\Delta\tau_i^N$ denotes $\tau_{i+1}^N - \tau_i^N$. As a first order equation, it is convenient to rearrange this as

$$\Delta\tau_n^N = -a^+(n)^{-1}\left(1 - a^-(n)(\Delta\tau_{n-1}^N)\right), \tag{16}$$

where $a^+(n) \neq 0$ for $0 \leq n \leq N$, and we impose $\Delta\tau_n = 0$ for $n < 0$. This is a first-order system and a general formula can be easily extrapolated. Indeed, we can verify by induction that this recursion is solved by,

$$\Delta\tau_n^N = -\sum_{i=0}^{n} \frac{[a^-(n)]^{\underline{i}}}{[a^+(n)]^{\underline{i+1}}}, \tag{17}$$

where for a one-variable function $f(x)$, we let $[f(x)]^{\underline{i}}$ denote $a(x)a(x-1)a(x-2)\cdots a(x-i+1)$. Now using the Fundamental Theorem of (discrete) Calculus, we have that,

$$\sum_{i=n}^{N-1} \Delta\tau_i^N = -\left((\tau_N^N - \tau_{N-1}^N) + (\tau_{N-1}^N - \tau_{N-2}^N) + \cdots + (\tau_{n+1}^N - \tau_n^N)\right) = \tau_N^N - \tau_n^N. \tag{18}$$

Observe that while N appears throughout, we have not yet used N at any point; however as $\tau_N^N = 0$ it follows that

$$\tau_n^N = -\sum_{i=n}^{N-1} \Delta\tau_i^N, \tag{19}$$

and then that

$$\tau_n^N = \tau_0^N - \tau_0^n. \tag{20}$$

Note that Equation (20) should be already expected for a memoryless system, as the time from 0 to N on average should be the time from 0 to n plus the time from n to N. Using Equation (19) and the formula in Equation (17) we have that,

$$\tau_n^N = \sum_{i=0}^{N-1}\sum_{j=0}^{i} \frac{[a^-(i)]^{\underline{j}}}{[a^+(i)]^{\underline{j+1}}} - \sum_{i=0}^{n-1}\sum_{j=0}^{i} \frac{[a^-(i)]^{\underline{j}}}{[a^+(i)]^{\underline{j+1}}}. \tag{21}$$

The dual case

Assume now that we are interested in the expected waiting time to reach a target N starting from copy number $n \geq N$, which we denote by τ_N^n. As the state-space is now unbounded (or one-sided), we may introduce an absorbing state $J \geq N$ such that no transitions out of J are allowed. This enables us to obtain an approximation to τ_N^n. The recurrence relation for τ_N^n continues to satisfy Equation (14). Finding a solution for τ_N^n is essentially the dual problem to solving τ_n^N, and so we can use the general procedure presented above to find a solution for τ_N^n, as we now explain. If we consider J to be the left-hand boundary and N to be the right-hand boundary (as opposed to 0 and N in the case for τ_n^N), we can make the following reinterpretation of the birth and death propensities. Defining $a^+(n) := a^-(J-n)$ and $a^-(n) := a^+(J-n)$, the general solution is now,

$$\tau_N^n = \sum_{i=0}^{J-N-1}\sum_{j=0}^{i} \frac{[a^-(i)]^{\underline{j}}}{[a^+(i)]^{\underline{j+1}}} - \sum_{i=0}^{J-n-1}\sum_{j=0}^{i} \frac{[a^-(i)]^{\underline{j}}}{[a^+(i)]^{\underline{j+1}}}. \tag{22}$$

Example 1: the simple birth-death process

Consider again the simple birth-death process introduced above in reaction scheme (1); an illustration of the model can be found in Figure 1(A). Here we provide details of the analytical solution (Equation (9) of the main text) used in the analysis given in the main text. From Equation (11) (alternatively Equation (7)

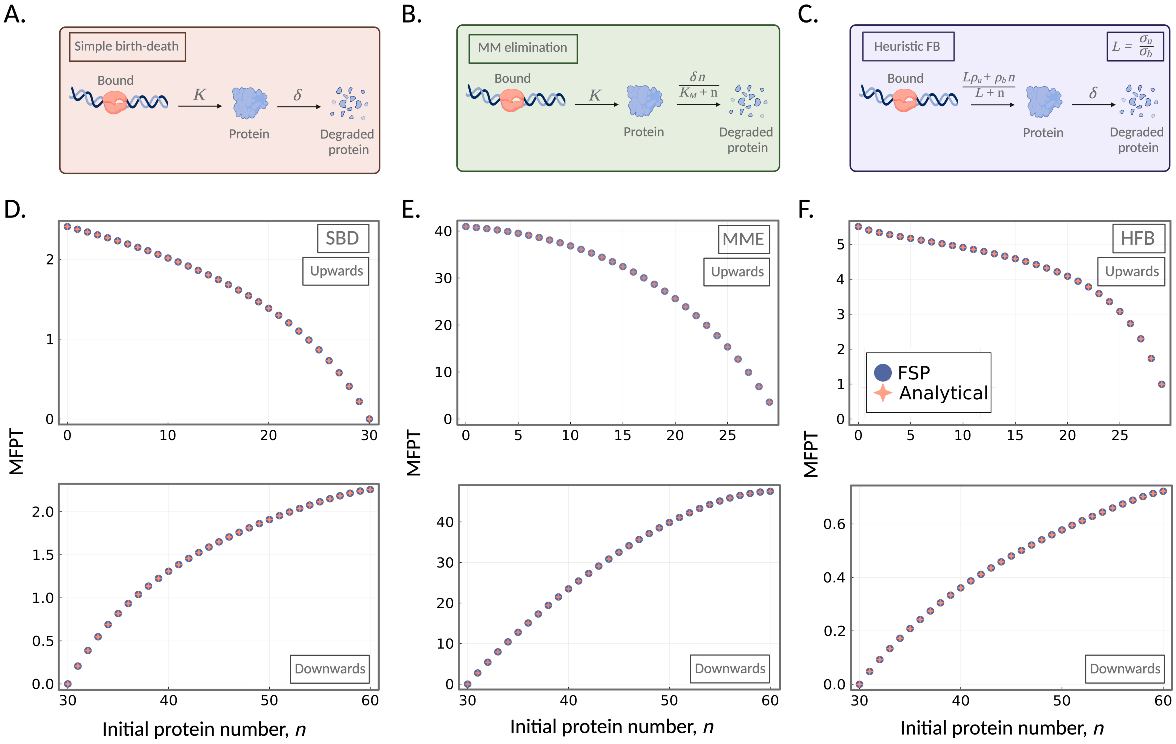

Supplementary Figure 1: A comparison of derived closed-form solutions for τ_n^N and τ_N^n of three birth-death models and the corresponding waiting times obtained from our FSP approach. (A) An illustration of the simple birth-death (SBD) process is given in the reaction scheme defined in Equation (1). (B) An illustration of the the birth-death process with Michaelis-Menten elimination (MME) given in reaction scheme (30). (C) An illustration of the heuristic feedback model defined in (33). (A)-(C) was created with BioRender.com. In (D)-(F) (top), we display the mean waiting time of the process to reach a target of $N = 30$ as a function of the initial protein number n $(0 \leq n \leq N)$; this is the upwards case. The analytical solutions (orange) are computed according to Equation (27), (32), and (35), respectively. The mean waiting times computed according to our FSP approach are shown in purple. In (D)-(F) (bottom), we consider the dual case, that is, the mean waiting time of the process to reach a target of $N = 30$ as a function of $n \geq N$; this is the downwards case. The parameters used in (D) for the SBD process are $K = 30$ and $\delta = 1$. For (E) the parameters used for the MME model are $K = 5$, $\delta = 6$ and $K_M = 5$. The parameters of the HFB model in (F) are $\sigma_u = 5$, σ_b=1, $L = \sigma_u/\sigma_b = 5$, $\rho_u = 10$, $\rho_b = 50$, and $\delta = 2$.

of the main text), the expected waiting time to reach N proteins given the system is started from $n \leq N$, can be described by the following recurrence relation,

$$(\delta n + K)\tau_n^N - \delta n \tau_{n-1}^N - K\tau_{n+1}^N = 1, \tag{23}$$

with $\tau_N^N = 0$ and base case at $n = 0$ of $K\tau_0^N - K\tau_1^N = 1$. From Equation (21) it follows that,

$$\tau_n^N = \frac{1}{K}\left(\sum_{i=0}^{N-1}\sum_{j=0}^{i} i^{\underline{j}}\left(\frac{\delta}{K}\right)^j - \sum_{i=0}^{n-1}\sum_{j=0}^{i} i^{\underline{j}}\left(\frac{\delta}{K}\right)^j\right). \tag{24}$$

We may remove the double sum by regrouping as a polynomial in terms of powers of $\frac{\delta}{K}$, which is equivalent to reversing the order of the sums. The coefficient of $\left(\frac{\delta}{K}\right)^m$ in Equation (24) can be seen to be $\sum_{j=0}^{N-1} j^{\underline{m}}$. This is usually written in terms of an equivalent expression involving the sum of rising factorials: $\sum_{j=1}^{N-m} j^{\overline{m}}$, which can be written as,

$$\sum_{j=1}^{N-m} j^{\overline{m}} = \frac{N^{\overline{m+1}}}{m+1}. \tag{25}$$

Thus we have for example,

$$\tau_0^N = \frac{1}{K}\sum_{i=0}^{N-1}\frac{N^{\underline{i+1}}}{i+1}\left(\frac{\delta}{K}\right)^i. \tag{26}$$

The solution for the mean first-passage time of the stochastic system is then given by,

$$\tau_n^N = \frac{1}{K}\left(\sum_{i=0}^{N-1}\frac{N^{\underline{i+1}}}{i+1}\left(\frac{\delta}{K}\right)^i - \sum_{i=0}^{n-1}\frac{n^{\underline{i+1}}}{i+1}\left(\frac{\delta}{K}\right)^i\right). \tag{27}$$

In the main text, we showed how the deterministic waiting time (Equation (11) of the main text) is a strict upper bound for Equation (27) when N is equal to $\rho K/\delta$ for $0 < \rho < 1$. On the other hand, we can see the deterministic waiting time as the limit, by finding, for any (small) $\varepsilon > 0$ a similar lower bound that converges to,

$$\frac{-\ln(1-(1-\varepsilon)\rho)}{\delta}. \tag{28}$$

To see this, take any positive $0 < \varepsilon < 1$ and consider the truncation of $\mathbb{E}(\tau_0)$ (Equation (27) with $n = 0$) by $i \leq \varepsilon N + 1$. As a truncated sum, this is a strict lower bound for the expected waiting time to $N = \rho K/\delta$ in the stochastic case. Observe that $N^{\underline{i+1}} \geq (N(1-\varepsilon))^{i+1}$, and replacing $N^{\underline{i+1}}$ in the truncated sum by $(N(1-\varepsilon))^{i+1}$ yields the lower bound,

$$\frac{1}{\delta}\sum_{i=0}^{\varepsilon N-1}\frac{((1-\varepsilon)\rho)^{i+1}}{i+1}, \tag{29}$$

which for any fixed ε (but in the limit of $K/\delta \to \infty$) yields the Taylor series for Equation (28) as claimed.

Example 2: Michaelis–Menten elimination

We now consider a birth-death process with Michaelis–Menten elimination. This prototypical reaction network has reactions,

$$\varnothing \underset{\sigma(n)}{\overset{K}{\rightleftharpoons}} P, \tag{30}$$

where K is a constant birth rate and the degradation $\sigma(n)$ is assumed to follow Michaelis-Menten kinetics $\frac{\delta n}{K_M+n}$; here n is the number of protein molecules in the system, δ is the degradation constant and K_M is the Michaelis-Menten constant. This model has been employed to capture so-called *active protein degradation* where proteins degrade according to some enzymatic reaction, as illustrated in Figure 1(B). Here the birth and death propensities are $a^+(n) = K$ and $a^-(n) = \sigma(n)$, and from Equation (11) (alternatively, Equation (7) in the main text), the expected waiting time to reach N from $n \leq N$ satisfies the recurrence relation,

$$\left(\frac{\delta n}{(K_M+n)} + K\right)\tau_n^N - \frac{\delta n}{(K_M+n)}\tau_{n-1}^N - K\tau_{n+1}^N = 1, \tag{31}$$

with $\tau_N^N = 0$ and base case at $n = 0$ of $K\tau_0^N - K\tau_1^N = 1$. From Equation (21), we have the following solution for the mean waiting time to reach N from $n \leq N$,

$$\tau_n^N = \frac{1}{K}\left(\sum_{i=0}^{N-1}\sum_{j=0}^{i}\frac{i^{\underline{j}}}{(K_M+i)^{\underline{j}}}\left(\frac{\delta}{K}\right)^j - \sum_{i=0}^{n-1}\sum_{j=0}^{i}\frac{i^{\underline{j}}}{(K_M+i)^{\underline{j}}}\left(\frac{\delta}{K}\right)^j\right). \tag{32}$$

Example 3: A reduced model of a feedback loop

Here, we consider one of the most prevalent heuristic stochastic model reduction approaches. As shown in Holehouse and Grima [2], in the limit of fast promotor switching, the stochastic feedback model (reaction scheme given in Equation (15) of the main text) reduces to just two reactions: an effective zero-order

reaction for the production of proteins, and a first-order reaction modelling protein degradation. The effective propensities for the two reactions are defined as follows.

$$a^{+}(n) := \frac{L\rho_u + \rho_b n}{L+n} \quad \text{and} \quad a^{-}(n) := \delta n, \tag{33}$$

where n is the number of proteins in the system and $L := \frac{\sigma_u}{\sigma_b}$. Refer to Figure 1(C) for an illustration of the model. From Equation (11) (alternatively Equation (7) of the main text), we obtain a tridiagonal recurrence relation for the mean waiting time τ_n^N of the heuristic model,

$$\left(\delta n + \frac{L\rho_u + \rho_b n}{L+n}\right)\tau_n^N - \delta n \tau_{n-1}^N - \left(\frac{L\rho_u + \rho_b n}{L+n}\right)\tau_{n+1}^N = 1, \tag{34}$$

with $\tau_N^N = 0$. From Equation (21), the mean waiting time to reach N starting from $n < N$ is given by,

$$\tau_n^N = \sum_{i=0}^{N-1}\sum_{j=0}^{i} \frac{(L+i)^{\underline{j+1}}}{(L\rho_u + i\rho_b)^{\underline{j+1,\rho_b}}} i^{\underline{j}}\delta^j - \sum_{i=0}^{n-1}\sum_{j=0}^{i} \frac{(L+i)^{\underline{j+1}}}{(L\rho_u + i\rho_b)^{\underline{j+1,\rho_b}}} i^{\underline{j}}\delta^j. \tag{35}$$

Turning to the deterministic regime, the reaction rate equation of the effective model in Equation (33) is given by,

$$\frac{dX}{dt} = \frac{L\rho_u + \rho_b X}{L+X} - \delta X, \tag{36}$$

with $X(0) = 0$. Solving for t gives,

$$t = \frac{1}{\mu^+ - \mu^-}\left[(\mu^+ + L)\log\left(1 - \frac{X}{\mu^+}\right) - (\mu^- + L)\log\left(1 - \frac{X}{\mu^-}\right)\right], \tag{37}$$

where $\mu^{\pm} := \frac{1}{2}\left(\rho_b - L \pm \sqrt{(\rho_b - L)^2 + 4L\rho_u}\right)$. Note that in [2], it has been shown that μ^+ is the steady-state mean of the deterministic system.

In Figure 1 (D)-(F), we verify the accuracy of the solutions given in Equation (27), (32), and (35), and their dual solutions, using comparisons with our FSP approach.

Supplementary Note 3 - Closed-form matrix solutions of FPT problems

In the previous section, we demonstrated how three-term (tridiagonal) recurrence relations arising from a birth-death process can be solved by way of the discrete forward derivative. Here we provide a more general approach that applies to any tridiagonal system of equations. Such systems of equations appear ubiquitously across the sciences, and many approaches have been developed to solve them; see for example [3], and more recently [4]. We illustrate below how our approach can be applied straightforwardly to solve systems with more that one state such as the telegraph model (refer to the reaction scheme given in Equation (13) of the main text, as well as the surrounding text for details of this model . Consider solving the following general tridiagonal recurrence relation,

$$C_{k-1}x_{k-1} + A_k x_k + B_k x_{k+1} = y_k, \tag{38}$$

for $k \in \{0, 1, 2, \dots\}$. Rewriting Equation (38) in matrix form, we have the following tridiagonal system of equations,

$$\begin{pmatrix} A_0 & B_0 & & & & & \\ C_0 & A_1 & B_1 & & & & \\ & C_1 & A_2 & B_2 & & & \\ & & C_2 & A_3 & B_3 & & \\ & & & \ddots & & & \\ & & & & & A_{n-1} & B_{n-1} \\ & & & & & C_{n-1} & A_n \end{pmatrix} \begin{pmatrix} x_0 \\ x_1 \\ x_2 \\ x_3 \\ \vdots \\ x_{n-1} \\ x_n \end{pmatrix} = \begin{pmatrix} y_0 \\ y_1 \\ y_2 \\ y_3 \\ \vdots \\ y_{n-1} \\ y_n \end{pmatrix}. \tag{39}$$

Using row operations, we can bring this into upper triangular form,

$$\begin{pmatrix} A_0' & B_0 & & & & & \\ & A_1' & B_1 & & & & \\ & & A_2' & B_2 & & & \\ & & & A_3' & B_3 & & \\ & & & \ddots & & & \\ & & & & & A_{n-1}' & B_{n-1} \\ & & & & & & A_n' \end{pmatrix} \begin{pmatrix} x_0 \\ x_1 \\ x_2 \\ x_3 \\ \vdots \\ x_{n-1} \\ x_n \end{pmatrix} = \begin{pmatrix} y_0' \\ y_1' \\ y_2' \\ y_3' \\ \vdots \\ y_{n-1}' \\ y_n' \end{pmatrix}, \tag{40}$$

where the new entries are related to the old ones by the following recursive relations,

$$A_0' = A_0 \qquad A_k' = A_k - C_{k-1}(A_{k-1}')^{-1}B_k, \qquad (k \geq 1) \tag{41}$$

$$y_0' = y_0 \qquad y_k' = y_k - C_{k-1}(A_{k-1}')^{-1}y_k' \qquad (k \geq 1). \tag{42}$$

Thus, we have reduced the original second-order system to two simpler recurrence relations of order one. The system can then be solved by back substitution,

$$x_n = (A_n')^{-1}y_n, \tag{43}$$

$$x_{n-1} = (A_{n-1}')^{-1}(y_{n-1} - B_{n-1}x_n), \tag{44}$$

$$x_{n-2} = (A_{n-2}')^{-1}(y_{n-2} - B_{n-2}x_{n-1}), \tag{45}$$

and so on. As we will see in the following example, there are many cases where the reduced system (43) can be solved straightforwardly by iteration.

The telegraph model

The state of the system is given by the number of mRNA and the gene state. We arrange our states as $(0, \text{off})$, $(0, \text{on})$, $(1, \text{off})$, $(1, \text{on})$, $(2, \text{off})$, ... Grouping them in blocks of two we obtain a block tridiagonal system with

$$A_k = \begin{pmatrix} k + \sigma_{\text{on}} & -\sigma_{\text{on}} \\ -\sigma_{\text{off}} & k + \sigma_{\text{off}} + \rho \end{pmatrix}, \tag{46}$$

$$B_k = \begin{pmatrix} & \\ & -\rho \end{pmatrix}, \tag{47}$$

$$C_k = \begin{pmatrix} -k & \\ & -k \end{pmatrix}. \tag{48}$$

$$\tag{49}$$

The entries A_k' satisfy

$$A_0' = \begin{pmatrix} \sigma_{\text{on}} & -\sigma_{\text{on}} \\ -\sigma_{\text{off}} & \sigma_{\text{off}} + \rho \end{pmatrix}, \tag{50}$$

$$A_k' = \begin{pmatrix} k + \sigma_{\text{on}} & -\sigma_{\text{on}} \\ -\sigma_{\text{off}} & k + \sigma_{\text{off}} + \rho \end{pmatrix} - \rho k (A_{k-1}')^{-1} \begin{pmatrix} & \\ & 1 \end{pmatrix} \qquad (k \geq 1). \tag{51}$$

We can verify by induction that this recursion is solved by

$$A_k' = \begin{pmatrix} k + \sigma_{\text{on}} & -k - \sigma_{\text{on}} \\ -\sigma_{\text{off}} & \sigma_{\text{off}} + \rho \end{pmatrix}. \tag{52}$$

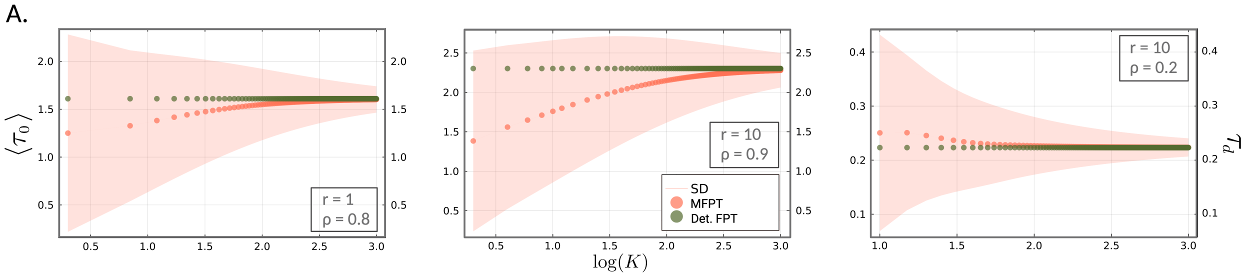

Supplementary Figure 2: Stochastic *vs.* deterministic waiting times to reach a target protein number for the bursty birth-death process with fixed burst size r. (A) FPTs under deterministic (green) and stochastic (orange) modelling regimes as a function of increasing K. The MFPT to reach a fixed target with $n = 0$ initial proteins, $\langle \tau_0 \rangle$, is computed according to Equation (9) in the main text, and the deterministic waiting time, τ_d, is computed according to Equation (11) in the main text. The standard deviation (SD) of the MFPT is given by the orange shaded area, and is again computed according to Equation (9) in the main text. Left panel: $\rho = 0.8$, K varies from 2 to 10^3, $\delta = 1$, and $r = 1$. Middle panel: $\rho = 0.9$, K varies from 2 to 10^3, $\delta = 1$, and $r = 10$. Right panel: $\rho = 0.2$, K varies from 10 to 10^3, $\delta = 1$, and $r = 10$.

It will be convenient to compute the determinants and inverses of these:

$$d'_k := \det A'_k = \rho(k + \sigma_{\text{on}}), \tag{53}$$

$$(A'_k)^{-1} = \frac{1}{\rho(k + \sigma_{\text{on}})} \begin{pmatrix} \sigma_{\text{off}} + \rho & k + \sigma_{\text{on}} \\ \sigma_{\text{off}} & k + \sigma_{\text{on}} \end{pmatrix}. \tag{54}$$

The right-hand side of the MFPT equation is changed to

$$y'_k = \left(\sum_{i=0}^{k} k(k-1)\dots(k-i)(A'_{k-i}A'_{k-i+1}\dots A'_{k-1})^{-1} \right) \begin{pmatrix} 1 \\ 1 \end{pmatrix}. \tag{55}$$

Our system of equations is thus

$$\begin{pmatrix} A'_0 & \begin{smallmatrix} 0 & 0 \\ 0 & -\rho \end{smallmatrix} & & & & & \\ & A'_1 & \begin{smallmatrix} 0 & 0 \\ 0 & -\rho \end{smallmatrix} & & & & \\ & & A'_2 & \begin{smallmatrix} 0 & 0 \\ 0 & -\rho \end{smallmatrix} & & & \\ & & & A'_3 & \begin{smallmatrix} 0 & 0 \\ 0 & -\rho \end{smallmatrix} & & \\ & & & \ddots & & & \\ & & & & & A'_{n-1} & \begin{smallmatrix} 0 & 0 \\ 0 & -\rho \end{smallmatrix} \\ & & & & & & A'_n \end{pmatrix} \begin{pmatrix} x_0 \\ x_1 \\ x_2 \\ x_3 \\ \vdots \\ x_{n-1} \\ x_n \end{pmatrix} = \begin{pmatrix} y'_0 \\ y'_1 \\ y'_2 \\ y'_3 \\ \vdots \\ y'_{n-1} \\ y'_n \end{pmatrix}. \tag{56}$$

Since we have the inverses of the A'_k, this can be computed straightforwardly using backsubstitution.

Supplementary Note 4 - Variability in FPTs

The bursty birth-death process with fixed burst size

Consider the bursty birth-death model with fixed burst size r defined in reaction scheme 8 of the main text; refer to Page 5 for a full description of the model. As expected, the variability in the FPT is highest when the discrepancy between the deterministic FPT and MFPT is largest, which occurs when molecule numbers are lowest; see Figure 2(A). This is observed across both high (left and middle) and low (right) targets.

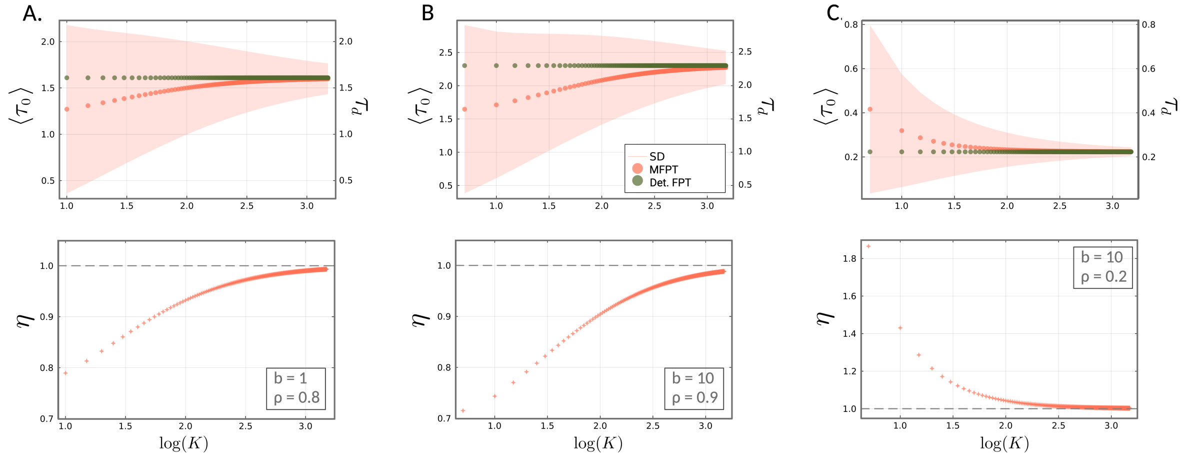

Supplementary Figure 3: Stochastic *vs.* deterministic waiting times to reach a target protein number for the bursty birth-death process with geometrically distributed burst sizes. (A-C) Top panel: The MFPT of the stochastic system (orange) to reach a fixed target with $n = 0$ initial proteins, $\langle\tau_0\rangle$, as a function of increasing K. The standard deviation (SD) of the MFPT is given by the orange shaded area. The corresponding deterministic waiting times, τ_d, are shown in green. Bottom panel: the corresponding ratio of the MFPT to the deterministic FPT, given by $\eta = \langle\tau_0\rangle/\tau_d$, as a function of increasing K. The model parameters are as follows: (A) $\rho = 0.8$, K varies from 10 to 1.5 x 10^3, $\delta = 1$, and $b = 1$. (B) $\rho = 0.9$, K varies from 1 to 1.5 x 10^3, $\delta = 1$, and $b = 10$. (C) $\rho = 0.2$, K varies from 1 to 1.5 x 10^3, $\delta = 1$, and $b = 1$.

The bursty birth-death process with geometrically distributed burst size

We next consider the bursty birth-death process where molecular species P is produced in bursts of size m. Here m is a random variable sampled from a geometric distribution $\phi(m) = p(1-p)^m$, where $p = \frac{1}{1+b}$ and b is the mean burst size. The reaction scheme is given by

$$\varnothing \xrightarrow{K} mP, \qquad P \xrightarrow{\delta} \varnothing. \tag{57}$$

By comparing the top panel of Figure 3 (A-C) with Figure 2(A), we see that the results for a bursty birth-death process with geometrically distributed burst sizes are qualitatively similar to those as for fixed burst sizes. Although in the case of a low target value ($\rho = 0.2$), we see a larger discrepancy in the MFPT and deterministic FPT when burst sizes are geometrically distributed. This can be seen by comparing the ratio plots given in Figure 3(C) (bottom) and Figure 2(F) in the main text.

The telegraph model

For the telegraph model described by reaction scheme 13 in the main text, we show how the variance changes as a function of the switching off rate μ, for three different values of λ; see Figure 4(A). For high and intermediate values ($\lambda = 10$ and $\lambda = 1$, respectively), we see that as μ increases, resulting in longer periods of gene inactivity, the variability in the FPT increases. However, for low values of λ ($\lambda = 0.1$), the variance is relatively constant as μ increases. These results are corroborated in (B) which shows the full (time-dependent) FPT distribution for the telegraph model, computed using Equation (13), for the three values of λ ($\lambda = 10$, $\lambda = 1$, and $\lambda = 0.1$). Additionally, we show (to the right of each distribution) the corresponding noise, as measured by the coefficient of variation (CV). The results reveal low (CV ≈ 0.27) to moderate (CV ≈ 0.68) noise in the distribution for high and intermediate values of λ. While the noise is higher (CV ≈ 1) for the case of low λ.

An autoregulatory feedback loop

Consider an autoregulatory feedback loop in which a protein produced by a gene either enhances or suppresses its own expression; for details of the model refer to reaction scheme 15 in the main text. Again we observe that the variability in the FPT (given by the orange shaded area) is highest when the discrepancy

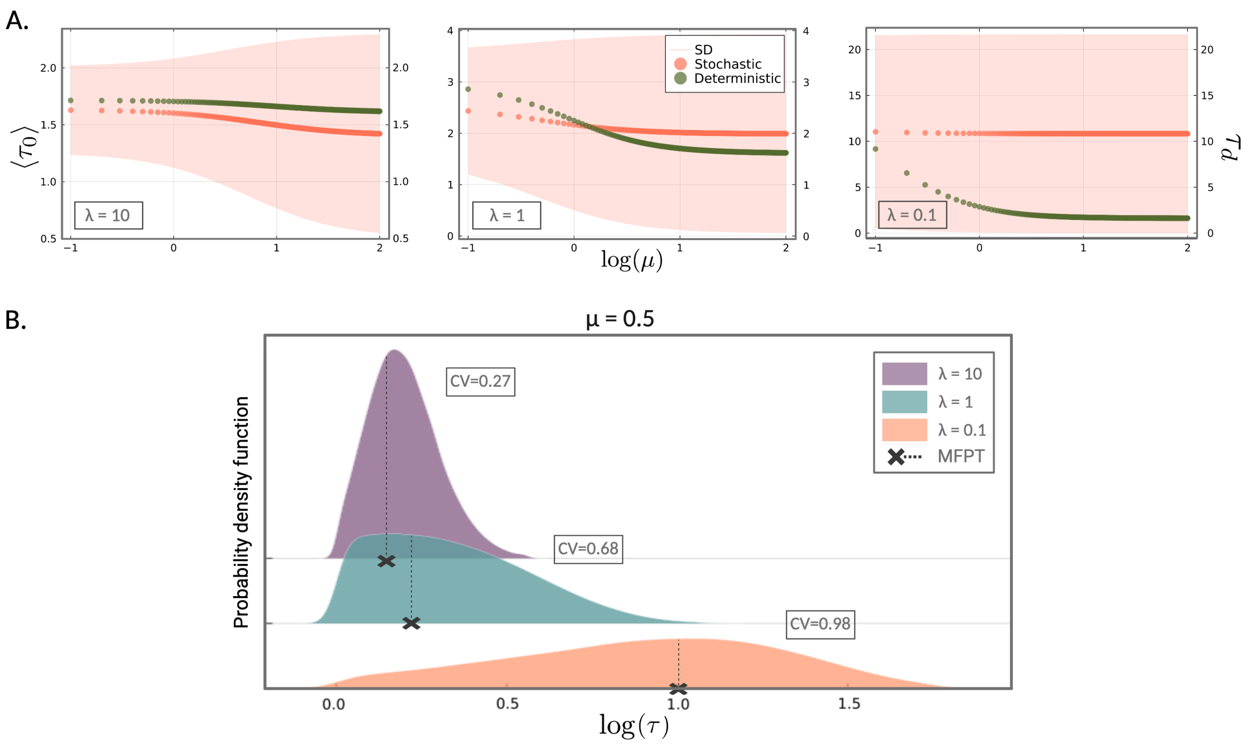

Supplementary Figure 4: Stochastic *vs.* deterministic waiting times to reach a target mRNA value for the telegraph model, as given by reaction scheme (13) in the main text. (A) Deterministic FPTs, τ_d (green), and stochastic MFPTs, $\langle\tau_0\rangle$ (orange), as a function of the switching off rate μ, for three different λ values: $\lambda = 10$ (left), $\lambda = 1$ (middle), $\lambda = 0.1$ (right). In each plot, we vary μ from 0 to 100, while keeping the steady-state mean mRNA number fixed at 100; this involves varying K accordingly. The target N is set to 80% of the steady-state mean. The standard deviation (SD) of the MFPT is given by the orange shaded area. (B) Time-dependent FPT distributions of the telegraph model for three different values of the switching-on rate λ considered in Figure 3(C) of the main text. The distribution for $\lambda = 10$ is shown in purple, $\lambda = 1$ in green, and $\lambda = 0.1$ in orange. In all cases, the switching-off rate $\mu = 0.5$, the transcription rate K is set so that the mean of the system is 100, $\delta = 1$, and the distribution is solved at $\tau = 100$. The MFPTs are shown as the grey crosses, and the coefficient of variation (CV) for each of the λ values is shown to the right of each distribution. The noise in the FPTs decreases as the switching rate increases, and the telegraph system becomes closer to a constitutive system.

between the deterministic FPT (plotted in green) and MFPT (plotted in orange) is largest, see Figure 5. This is observed for both positive (A) and negative (B) feedback.

Limiting cases

For all models considered so far, the FPT prediction of the deterministic model falls within one standard deviation (SD) of the MFPT of the stochastic model. Here we consider cases where the deterministic prediction falls well outside of this range. As mentioned in the introduction of the main text, if for a given initial condition, the target molecule number is outside of the range predicted by the deterministic model in finite time, the latter's prediction for the mean time is undefined, whereas a stochastic model will typically predict a finite value. In these cases, the deterministic model only approaches the target asymptotically. Therefore, by choosing a target that is very close to the steady-state mean number of molecules, we expect the FPT prediction of the deterministic model to be well outside of one, or even two, SDs of the MFPT. In Figure 6 we demonstrate this with a model of the simple birth-death process. Here, we consider a target very close to the steady-state mean number of molecules ($\rho = 0.999$) and indeed the deterministic FPTs lie outside one SD of the mean (the orange shaded region). We emphasize that this is independent of the choice of model.

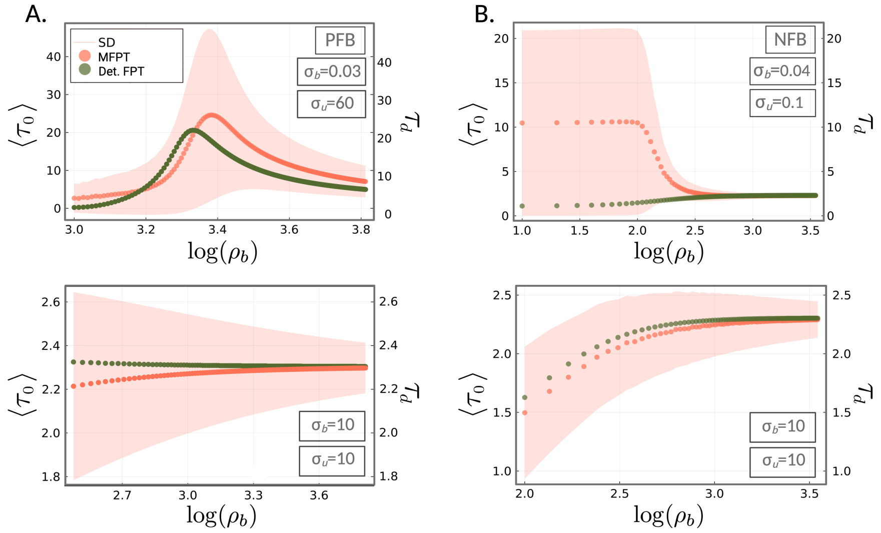

Supplementary Figure 5: Stochastic *vs.* deterministic waiting times to reach a target protein value for auto-regulatory feedback models. The MFPT of the stochastic system (orange) to reach 90% of the steady-state mean, given the system is started from 0 protein molecules, $\langle \tau_0 \rangle$, for increasing ρ_b. The standard deviation (SD) of the MFPT is given by the orange shaded area. The corresponding deterministic waiting times, τ_d, are shown in 8green. (A) A positive auto-regulatory feedback model with parameters: $\rho_u = 10$, ρ_b varies from 1000 to 6500, $\sigma_b = 0.03$, and $\sigma_u = 60$ (top panel); $\rho_u = 10$, ρ_b varies from 300 to 6500, $\sigma_b = 10$, and $\sigma_u = 10$ (bottom panel). (B) A negative auto-regulatory feedback model with parameters: $\rho_u = 3500$, ρ_b varies from 10 to 3500, $\sigma_b = 0.04$, and $\sigma_u = 0.1$ (top panel); $\rho_u = 3500$, ρ_b varies from 100 to 3500, $\sigma_b = 10$, and $\sigma_u = 10$ (bottom panel).

Supplementary Note 5 - Computational efficiency of the modified FSP

In this section, we present three applications of our FSP approach for computing MFPTs. We demonstrate the versatility of the approach by applying the method to three exemplary models from the literature: (1) a birth-death process with Michaelis-Menten degradation; (2) a compartmental model of disease spread; and, (3) a Michaelis-Menten reaction scheme. Our approach is shown to be significantly more computationally efficient than traditional Monte-Carlo simulations. All simulations were run on a 2.6 GHz 6-Core Intel Core i7 processor with 32GB RAM.

Example 1: A birth-death model with Michaelis–Menten degradation

Consider again the simple birth-death system with Michaelis–Menten degradation given in Equation (30) above (see Figure 7(A)). We are interested in the time that it takes for the protein number n to reach a certain threshold value N. Here, we set N to be the steady-state mean of the system, and consider the mean waiting time of the system to reach this threshold, conditional on the system starting from some initial state $\mathbf{n}_0$. This information provides insights into the underlying biochemical processes and the timescales associated with protein-enzyme interactions.

In Figure 7(B), we display a representative time series of the protein number n. The vertical orange lines indicate the times at which the protein count is $n = 0$ and when it has reached the steady-state mean ($N = 30$). From the single trajectory presented, we observe that it takes approximately 78 time units for the protein count to first reach the threshold $N = 30$, given the number of proteins in system is initially $n = 0$. As this is a stochastic process, the mean of the first passage time is a more representative measure of the process dynamics. One advantage of our approach for computing mean first passage times is that it allows us to simultaneously determine the MFPT for all initial protein counts that are less than the threshold value. In Figure 7(C), we plot the mean waiting time to reach the steady-state value ($N = 30$) for different initial protein numbers. Here we let the initial protein $\mathbf{n}$ vary between 0 and 30. As expected, the lower the initial protein count, the longer the system takes to reach the threshold value.

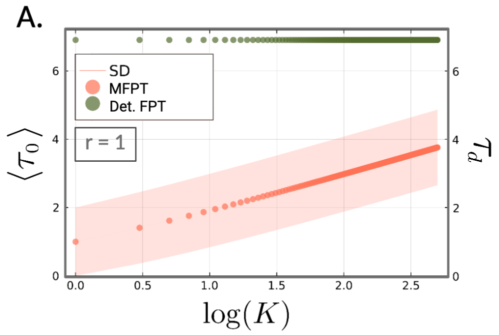

Supplementary Figure 6: Stochastic *vs.* deterministic waiting times to reach a target protein number for the bursty birth-death process with fixed burst size r: a limiting case. (A) The MFPT of the stochastic system (orange) to reach a fixed target with $n = 0$ initial proteins, $\langle \tau_0 \rangle$, as a function of increasing K. The standard deviation (SD) of the MFPT is given by the orange shaded area. The corresponding deterministic waiting times, τ_d, are shown in green. The model parameters are as follows: K varies from 1 to 500, $\delta = 1$, $\rho \approx 1$, and $r = 1$.

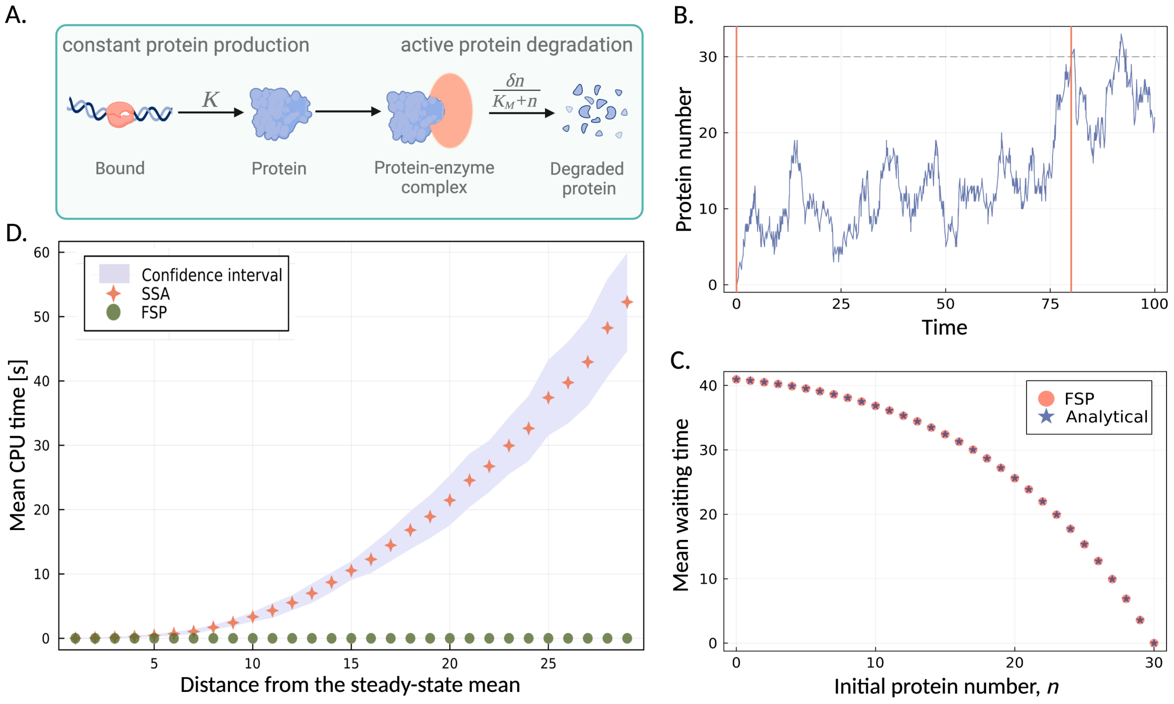

Supplementary Figure 7: Computational cost of the FSP approach to compute the MFPT distribution using a one dimensional birth-death system with Michaelis–Menten (MM) degradation kinetics. (A) An illustration of a birth-death system with MM degradation kinetics. Protein is produced constantly at rate K and actively degraded by an enzymatic reaction with rate $\delta n/(K_M + n)$. (A) was created with BioRender.com. (B) The protein number as a function of time. The system is initialised at $n = 0$ (first orange vertical line) and stopped when n first reaches the threshold value of $N = 30$. The grey dotted line represents the threshold value and the second orange vertical line indicates the first time the system reaches the threshold value. (C). The mean waiting time as a function of the initial protein number. (D) Comparison of the CPU time taken to calculate the MFPT distribution using FSP (orange) and the SSA (green) as a function of the initial protein number. The purple shading indicates the 95% confidence interval. The model parameters used are: $K_b = 5$; $K_d = 6$; $K_M = 5$. The SSA is run for the number of trajectories it takes for the MFPT to agree with the MFPT given by the FSP, with an error tolerance of 10%.

We next compare the computational time of computing MFPTs using our FSP approach with that of the

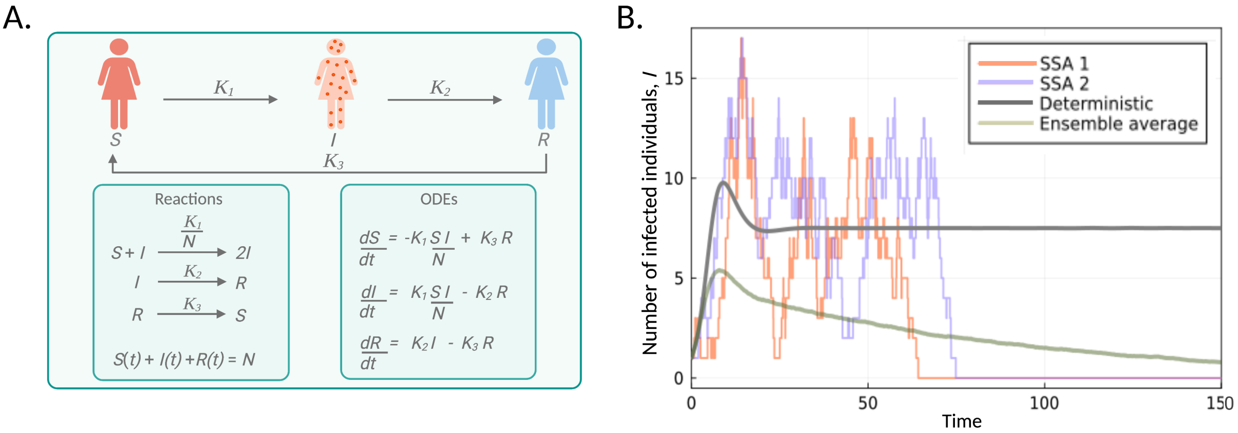

Supplementary Figure 8: The deterministic and stochastic dynamics of an epidemic SIRS model. (A) An illustration of the SIRS model where the population is divided into susceptible (S), infected (I) and recovered (R) individuals. The typical trajectory is for an individual to move from S to I to R, where individuals in state R can again move to state S due to a waning immunity. The chemical reactions are given in the left-hand side box and the associated reaction rate equations are shown in the right-hand side box. (A) was created with BioRender.com. (B) The deterministic model (grey curve) predicts that the number of infected individuals in a population reaches a non-zero steady-state. Stochastic trajectories obtained using the SSA (orange and purple curves), however, are driven to the extinction state by random fluctuations. The ensemble average of 5 x 10^3 stochastic trajectories is shown in the green curve and differs distinctly from the deterministic prediction.

stochastic simulation algorithm (SSA). Figure 7(D) shows the mean processing time required to obtain the MFPT conditional on the initial protein number, using the stochastic simulation algorithm (shown in orange) and our FSP approach (shown in green). The computation time for the MFPTs shown in Figure 7(C) is on average 0.26 milliseconds when using the FSP approach, and is approximately 53 seconds when using the SSA. This difference in computation time is due to the fact that the FSP approach allows for the calculation of the MFPT for *all* initial states $\mathbf{n}$ in a single computation, whereas the SSA requires the computation of multiple trajectories for each initial condition $\mathbf{n}$ in order to construct the MFPT curve given in Figure 7(C).

Example 2: An epidemic SIRS model

We here consider one of the simplest compartmental models in epidemiology: the SIRS model [5, 6]. Here the population is partitioned into three compartments: Susceptible (S), Infected (I), and Recovered (R) individuals. The total population size, N, is fixed such that $S + I + R = N$. Individuals in state S are considered to be at risk of contracting the disease, but are currently uninfected. Individuals in state I have been infected with the disease and are capable of transmitting it to others. Individuals in state R are recovered, but are able to become susceptible again – modelling a waning immune response.

The SIRS model is defined by the following reaction equations,

$$S + I \xrightarrow{K_1/N} 2I, I \xrightarrow{K_2} R, R \xrightarrow{K_3} I, \tag{58}$$

where K_1/N is the transmission rate, K_2 is the rate of recovery and K_3 is the reinfection rate. An illustration of the SIRS model is shown in Figure 8(A). The basic reproduction number, $\mathfrak{R}_0$, is equal to K_1/K_2; this ratio is derived as the expected number of new infections from a single infection in a population where all subjects are susceptible. Note that when solving Equation (58), we exploit the conservation law $S = N - R - I$, giving us an effective two-compartmental model in I and R. The associated deterministic equations of the two-compartmental model are,

$$\frac{dI}{dt} = \frac{K_1(N - I - R)I}{N} - K_2 I, \tag{59}$$

$$\frac{dR}{dt} = K_2 I - K_3 R. \tag{60}$$

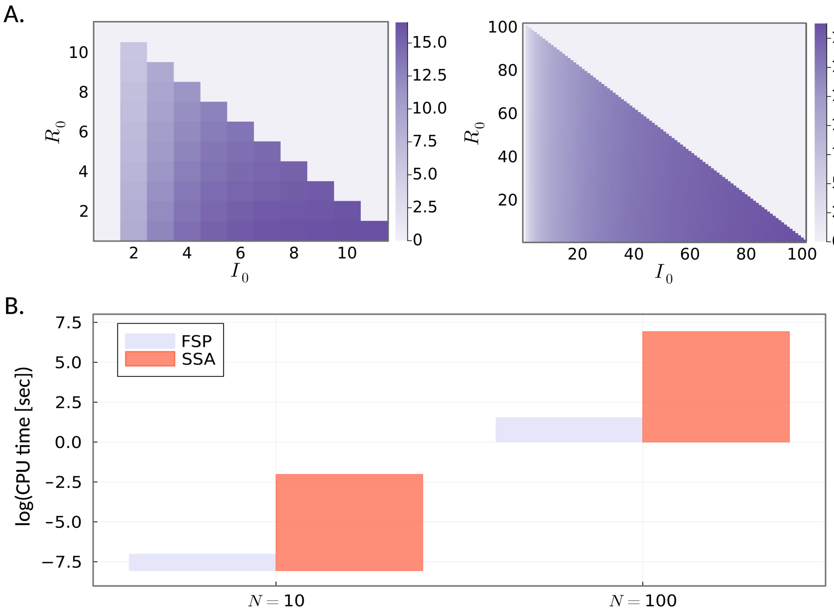

Supplementary Figure 9: Computational cost of the FSP approach to compute the MFPT distribution using an epidemic SIRS model of infection. (A) Heatmaps showing the mean time to extinction as a function of the parameters I_0, the initial number of infected individuals, and R_0, the initial number of recovered individuals, for a population size of $N = 10$ (left panel) and $N = 100$ (right panel). (B) Comparison of the median CPU time of the FSP approach (purple) and the SSA (orange) as a function of the population size N. Note that for $N = 10$, we re-normalise the baseline CPU time for visualisation purposes. Parameters used: $K_1 = 0.5$, $K_2 = 0.5$, $K_3 = 0.3$, $N = 10$ (left panel). $K_1 = 0.05$, $K_2 = 0.3$, $K_3 = 0.3$, $N = 100$ (right panel).

When the basic reproduction number $\mathcal{R}_0 > 1$, the deterministic regime realises a so-called *endemic equilibrium* (a non-zero steady-state), and, as such, predicts that the disease will never die out. In comparison, the stochastic regime is able to converge to the disease-free state. We illustrate this behaviour in Figure 8(B), where we plot the number of infected individuals over time. Trajectories of the SIRS model simulated using the SSA fluctuate around the deterministic steady-state mean for a period of time, before fluctuations drive the trajectories to zero (orange and blue trajectories). The stochastic ensemble average (green curve) converges to zero and thus disagrees with the deterministic prediction (grey curve). As such, it is necessary to turn to a stochastic framework when modelling the extinction time of an infectious disease. Using a stochastic framework, we can model the duration of the epidemic as a first passage time problem.

We define the extinction time as the expected waiting time of the system in Equation (58) to reach zero infected individuals, given it was initialised with a pre-specified number of infected (I_0), susceptible ($S_0 = N - I_0 - R_0$), and recovered (R_0) individuals. We are interested in computing the mean time to extinction only in the stochastic regime since the deterministic regime cannot achieve disease extinction. In Figure 9(A) we plot heatmaps of the extinction time, as a function of the initial number of infected I_0 and recovered R_0 individuals, for population size $N = 10$ and $N = 100$, using our FSP approach. The extinction time is greatly affected by the initial number of infected individuals. The larger the proportion of infected individuals is at the start of the epidemic, the longer it takes the epidemic to die out, and vice-versa. It is well-appreciated that the time to extinction, in SIR(S) models, is profoundly affected by the both the initial number of infected individuals and the size of the population [7].

Next, we are interested in the average CPU time taken to achieve the results in Figure 9(A), using our FSP approach and the SSA. Figure 9(B) shows the median CPU time in seconds for $N = 10$ and $N = 100$ for both the FSP (orange) and SSA (purple). The FSP is considerably more time efficient than the SSA, approximately four orders of magnitude when $N = 10$ and seven orders of magnitude when $N = 100$. Reasoning for the reduction in computational time follows the same logic as the birth-death model with MM degradation.

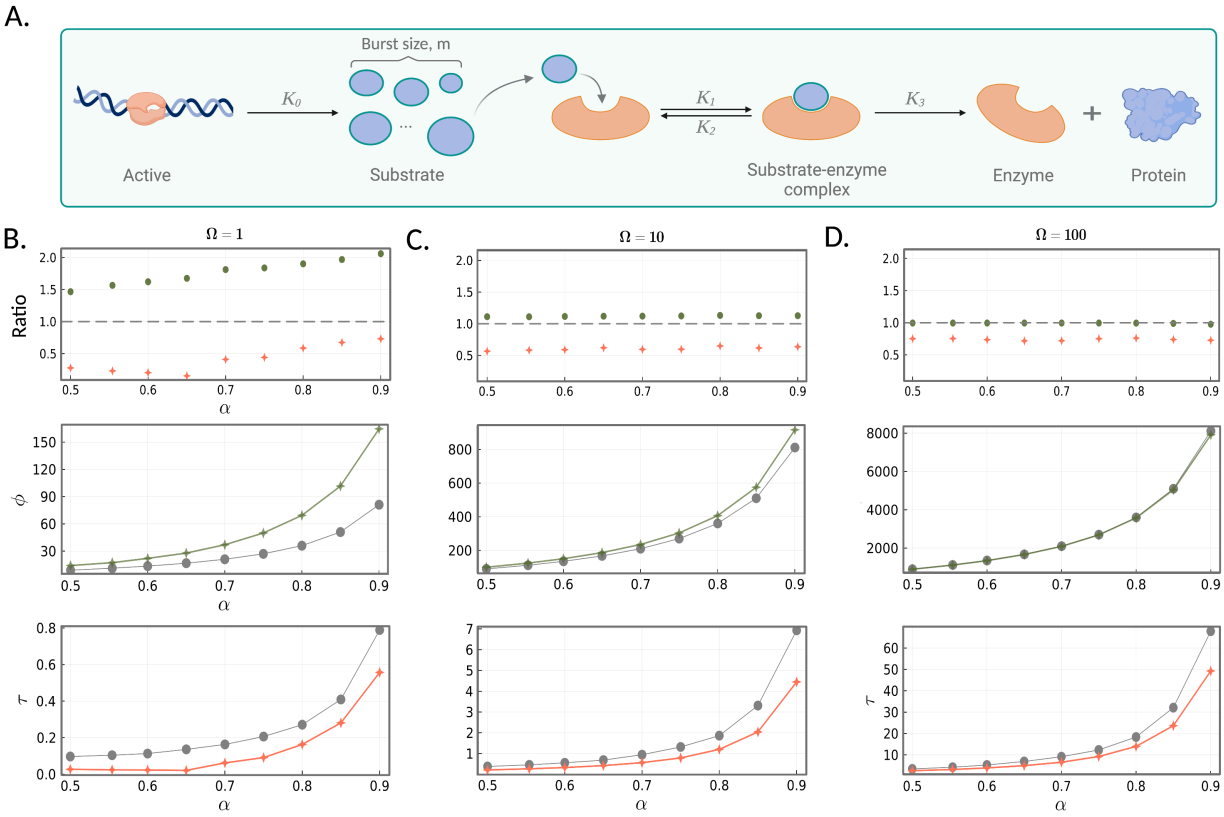

Supplementary Figure 10: The Michaelis-Menten equation in steady-state conditions. (A) An illustration of the Michaelis-Menten reaction mechanism given by the reaction scheme in (61); this was created with BioRender.com. In (B)-(D) (top panels) we plot the substrate ratios $\varphi_{\text{sto}}/\varphi_{\text{det}}$ (green circles) and the associated waiting time ratios $\tau_{\text{sto}}/\tau_{\text{det}}$ (orange stars), as a function of the ratio α (a measure of the enzyme saturation). In the middle panels, we compare φ under deterministic (grey) and stochastic (green) models. In the bottom panels we compare τ under deterministic (grey) and stochastic (orange) models, as a function of α. (B) When the volume Ω is 1, the discrepancy between φ and τ is largest, and increases as α approaches 1. As the volume Ω increases, from (C) 10 to (D) 100, the discrepancy decreases. Model parameters used: $K_1 = 4$, $K_2 = 3$, $K_3 = 37$, $E_T = 60$ and $\Omega \in \{1, 10, 100\}$. α is varied through K_0.

Example 3: Michaelis-Menten kinetics in steady-state conditions

We consider a generalised stochastic model of the well-studied Michaelis-Menten reaction mechanism [8], where the enzyme kinetics are confined to a sub-cellular compartment of volume Ω. The reactions are given by,

$$\varnothing \xrightarrow{K_0} mS, \quad S + E \underset{K_2}{\overset{K_1}{\rightleftharpoons}} ES \xrightarrow{K_3} E + P. \tag{61}$$

Here, a substrate molecule S is spontaneously created in bursts and continuously supplied to the sub-cellular compartment at rate K_0. In the sub-cellular compartment, S reversibly binds to a free enzyme molecule E with rates K_1 and K_2, respectively, to form the complex ES. The complex subsequently yields a product molecule P, with rate K_3. The parameter m represents the burst size, which for simplicity, we take here to be a constant (see Figure 10(A)). The total number of enzyme molecules E_T is conserved such that $E_T = E + ES$. The system has a steady state in S if $\alpha = \frac{mK_0\Omega}{K_3 E_T} \leq 1$, in other words, if the supply rate of S is less than the maximum production rate of P. In this way, the ratio α provides a measure of how saturated the enzyme is with substrate. Note that $\alpha > 1$ implies that the substrate numbers increase unboundedly with time.

We assess the validity of the deterministic description of the enzyme kinetics by comparing to exact stochastic simulations of the MM reaction scheme (Equation (61)). Specifically, we are interested in the

steady-state concentration of S, and the associated mean waiting time to reach the steady-state, given that the system is started with zero substrate and all enzyme initially in the unbound, free state. Let φ be 90% of the steady-state mean substrate concentration and τ be the expected waiting time of the system to reach φ. To obtain φ in the deterministic regime, we solve the deterministic reaction rate equations at steady-state. The associated expected waiting time τ is computed by solving the rate equations and recording the first time that S reaches φ. In the stochastic regime, we obtain φ by way of SSA. We take the average over 1000 independent simulations of Equation (61) for a time-span of 10^3, collecting every 20 time units. We utilise our FSP approach to compute the associated mean waiting time, τ. All simulations are performed in the Julia Programming Language [9].

In Figure 10(B)-(D), we examine the effect of a sub-cellular compartmental volume on both φ and the associated mean waiting time τ, between the stochastic and deterministic regimes. The two regimes are compared for increasing volumes, $\Omega \in \{1, 10, 100\}$. In the top panels, we consider the substrate ratio, given as $\varphi_{\text{sto}}/\varphi_{\text{det}}$, and the expected-waiting-time ratio, given as $\tau_{\text{sto}}/\tau_{\text{det}}$, as a function of the saturation constant α. Note that when the deterministic and stochastic regimes are in agreement, the value of these ratios is unity. In the low molecular regime (B) & (C), the stochastic simulations consistently yield higher substrate concentrations than those predicted by deterministic rate equations. This discrepancy becomes more significant as α increases, and can also be observed in the remaining plots, which illustrate the dependence of φ (middle panel) and τ (bottom panel) on α. As α approaches unity, the system becomes unstable, and the fluctuations around the mean substrate concentration become increasingly large, causing significantly higher φ values in the stochastic regime. This results in an infinite ratio between the two regimes in the limit. Conversely, the waiting time for the system to reach φ is consistently lower in the stochastic regime, despite higher substrate concentrations. This can be seen in the ratio plots (top panel), where the mean waiting time ratio is always below one, despite the substrate ratio consistently being greater than or equal to one. The bottom panel plots clearly show that the waiting time τ in the stochastic regime (orange stars) is always less than the deterministic regime (grey dots). Thus, increasing molecular noise in the system (by decreasing the system size Ω) decreases the waiting time to reach φ, implying that intrinsic noise profoundly affects the mean first-passage time. For larger systems (Figure 10(D)), we observe convergence between the stochastic and deterministic substrate concentrations. However, we can see that the waiting times converge significantly more slowly, requiring a much larger system size for convergence.

Supplementary Table 1 - Parameters for Figure 4 of the main text

Model parameters used in Figure 4 of the main text

**Fig. 4.**	**Panel**	**Parameters**				**Mean**	
		σ_b	σ_u	ρ_b	ρ_u	Deterministic	Stochastic
(B)	Top and middle	0.03	60	300–6500	10	11.68–4504.44	11.68–4487.80
	Bottom	0.03	0.1–60	500–6500	10		
(C)	Top and middle	10	10	300–6500	10	300–6499.01	299.07–6498.60
	Bottom	10	0.1–6	300-6500	10		
(D)	Top and middle	0.04	0.1	10–3500	3500	97.36–3500	44.36–3499.90
	Bottom	0.04	0.1–10	10–1000	1000		
(E)	Top and middle	10	10	100–3500	3500	126.64–3500.64	126.24–3500
	Bottom	10	0.1–10	100–1000	1000		

Supplementary References

[1] N. G. Van Kampen, Stochastic Processes in Physics and Chemistry, Elsevier, 1992.

[2] J. Holehouse, R. Grima, Revisiting the reduction of stochastic models of genetic feedback loops with fast promoter switching, Biophys. J. 117 (2019) 1311–1330.

[3] H. Risken, The Fokker-Planck equation, Springer, 1996.
[4] J. Holehouse, Closed-form solution of a general three-term recurrence relation: applications to heun functions and social choice models, arXiv preprint arXiv:2302.04187 (2023).
[5] H. W. Hethcote, An immunization model for a heterogeneous population, Theor. Popul. Biol. 14 (1978) 338–349.
[6] H. W. Hethcote, S. A. Levin, Periodicity in epidemiological models, in: S. A. Levin, T. G. Hallam, L. J. Gross (Eds.), Applied Mathematical Ecology, Springer Berlin Heidelberg, Berlin, Heidelberg, 1989, pp. 193–211.
[7] L. J. Allen, Stochastic population and epidemic models, Mathematical biosciences lecture series, stochastics in biological systems (2015) 128.
[8] R. Grima, An effective rate equation approach to reaction kinetics in small volumes: Theory and application to biochemical reactions in nonequilibrium steady-state conditions, J. Chem. Phys. 133 (2010) 035101.
[9] J. Bezanson, A. Edelman, S. Karpinski, V. B. Shah, Julia: A fresh approach to numerical computing, SIAM review 59 (2017) 65–98.
